# A Rheological Model for Evaluating the Behavior of Shear Thickening of Highly Flowable Mortar

**DOI:** 10.3390/molecules26041011

**Published:** 2021-02-14

**Authors:** Mengyuan Li, Jianguo Han, Yuqi Zhou, Peiyu Yan

**Affiliations:** 1 Department of Civil Engineering, Tsinghua University, Beijing 100084, China; limengyu15@tsinghua.org.cn (M.L.); hanjg@mail.tsinghua.edu.cn (J.H.); zhouyuqi@chinaonebuild.com (Y.Z.); 2 China Construction First Group Construction & Development Co. Ltd., Beijing 100102, China

**Keywords:** shear thickening, highly flowable mortar, exponential rheological model, yield stress, differential viscosity, consistency exponent

## Abstract

Neither the modified Bingham model nor the Herschel–Bulkley model can be used to characterize and calculate the performance of shear thickening of highly flowable mortar because of their incalculability of the rheological parameters. A new exponential rheological model was established to solve the characterization and calculation of shear thickening of the lubrication layer (highly flowable mortar) during the pumping of concrete in this paper. This new exponential rheological model has three rheological parameters, namely, yield stress, consistency coefficient, and consistency exponent. They can quantitatively describe the yield stress, differential viscosity, and shear thickening degree of highly flowable mortar. The calculating results of the rheological parameters of the newly established model for the mortars with different compositions showed that the consistency exponent of mortar decreased with the increase of its sand-binder ratio or the dosage of fly ash in the binder. This indicates that the shear thickening degree of mortar decreases. The consistency exponent of mortar initially decreases and subsequently increases with the increase in silica fume content or the dosage of the superplasticizer. It illustrates that the degree of the shear thickening of mortar initially decreased and subsequently increased. These varying patterns were confirmed by the rheological experiment of mortars.

## 1. Introduction

Nowadays, the pumping operation is the most popular transportation process of concrete in the construction of all kinds of buildings. In order to ensure the safety of the pumping operation, the pumping performance of concrete should be evaluated aforehand. A large number of studies [1,2,3] have shown that the pumping performance of concrete is closely related to the performance of the lubrication layer, which is a paste-rich mortar layer formed near the pipe wall during the pumping process. In order to transport concrete safely and smoothly, a highly flowable concrete is used. Therefore, the lubrication layer is regarded as highly flowable mortar.

During the pumping process, the pump pressure is very high, which makes the lubrication layer bear quite high shear stress. In this case, the lubrication layer tends to have significant shear thickening characteristics. The effect of shear thickening must be considered in the case of high shear stress such as pumping and strong agitation [4,5]. The Kaplan model is the commonly utilized model to evaluate the relationship between pump pressure and flow rate in the pumping process [6,7]. The establishing process of this model supposes that pumped concrete conforms to the Bingham model and the shear thickening effect is neglected. At present, there is still a considerable gap between the pumping performance of concrete predicted by the model and the data obtained in the actual pumping process, which fully indicates the importance and necessity of considering the shear thickening effect under high shear stress [8,9]. How to correctly characterize and calculate the rheological parameters of highly flowable mortar under high shear stress is the basis and premise to accurately evaluate the pumping performance of highly flowable concrete.

In addition to the research on the properties of thixotropic elasto-visco-plastic materials [10,11], the study on the shear thickening performance of fluid is also one of the main research interests of rheologists. For example, the constitutive model characterizing the shear thickening performance of suspensions under the condition of superposed transverse oscillations has been studied in the literature [12]. The constitutive model for time-dependent flows of shear-thickening suspensions has been established in the literature [13]. The pumping process of highly flow concrete is a time-independent process without transverse oscillations because the pumped concrete is usually mixed with an appropriate amount of retarder. The modified Bingham model and Herschel–Bulkley model are commonly used rheological models to characterize the shear thickening performance of fresh cementitious materials. Although the modified Bingham model seems to be able to represent the shear thickening effect of highly flowable mortar in form, it violates the essence of the shear thickening effect [14] and it is very difficult to calculate the rheological parameters [15]. Therefore, this model is not suitable for the characterization and calculation of the rheological parameters of cementitious materials in the worksite. The rheological parameters of the Herschel–Bulkley model cannot be calculated [15] and can only be used for theoretical research and analysis. Therefore, it cannot be used to evaluate the performance of pumped concrete and the lubrication layer in practical engineering. The rheological determination of time dependent suspensions such as cementitious pastes remain a struggle and difficult task due to thixotropy, time dependency (hydration), settlement due to gravity, and the migration of particles depending on device, slip, and operator handling. Nevertheless, in-line measurements can facilitate the task and it have already been successfully applied in the measurements of the rheological properties of cementitious materials [16,17]. It is necessary to establish a new rheological model that not only conforms to the essence of the shear thickening effect of highly flowable mortar, but also facilitates the measurement and calculation of rheological parameters in the worksite.

## 2. Literature Study

### 2.1. Theory of Shear Thickening

High flowable mortar and concrete often show the performance of shear thickening rather than shear thinning under the action of high shear stress [14,18,19]. There are two famous theories to explain the phenomenon of shear thickening. One is cluster formation, and another is grain inertia.

#### 2.1.1. Cluster Formation

**Figure 1:**
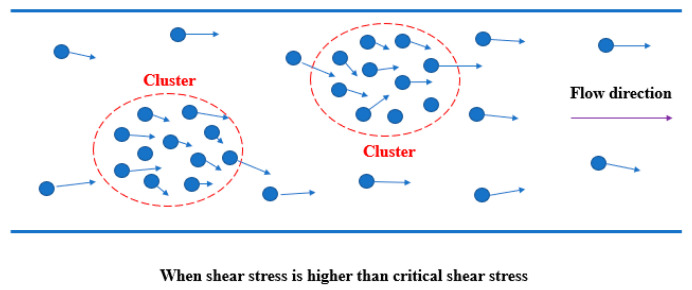
Schematic diagram of cluster formation theory.

The theory of cluster formation considers that the phenomenon of shear thickening contributes to the formation of clusters [14,20]. When shear stress exceeds the critical ones [21,22], the high hydrodynamic forces among the dispersed particles in the fluid can overcome the repulsive force, which includes Brownian force, electrostatic force, spatial force, etc. Then, a temporary assembly of particles named as the cluster forms. When the critical shear stress [21,22] is exceeded, the ratio of the hydrodynamic force to the repulsive force is greater than 1 and the relaxation time of the repulsive force is much longer compared with that of the hydrodynamic force. This results in the retention of particles in the clusters. Particles can join or leave the cluster, so the cluster is a temporary phenomenon (Figure 1). If a cluster is formed in the shear zone, a certain degree of blockage occurs, which makes the flow unsmooth. This blockage increases the viscosity by a large amount, resulting in a shear thickening. If the applied shear stress is less than the critical shear stress, the repulsive force dominates the hydrodynamic force and the clusters disappear. Therefore, shear thickening is completely reversible.

The Peclet number in Equation (1) is used to measure the relative magnitude of hydrodynamic viscosity under the hydrodynamic force and Brownian viscosity under the repulsive force [14]. Brownian viscosity is dominant when the value of the Peclet number is lower than 1. Brownian viscosity gradually decreases and hydrodynamic viscosity gradually dominates with the increase in Peclet number. When the Peclet number reaches the magnitude of 10^3^, the Brownian viscosity hardly matters.

(1) $$Pe=\frac{\eta_{s}\cdot \dot{\gamma }\cdot a^{3}}{k\cdot T}$$

where *Pe* is the Peclet number (-); $\eta_{s}$ is apparent viscosity of suspending medium (Pa·s); $\dot{\gamma }$ is shear rate (1/s); *a* is particle radius (m); *k* is Boltzmann’s constant (1.38 × 10^−23^ J/K); and *T* is temperature (K).

#### 2.1.2. Grain Inertia

The theory of grain inertia holds that shear thickening is caused by momentum transfer among suspended particles [20]. Momentum can be transferred directly from particle to particle, which is the characteristic of grain inertia. Reynolds number (*Re_P_*) in Equation (2) describes the ratio of inertia to viscous force [20]. If the value is far less than 0.1, the inertia is completely controlled by viscous force, and the effect of grain inertia can be ignored. If the value is large, the effect of particle inertia cannot be ignored.

(2) $$Re_{P}=\frac{\rho_{s}\cdot \dot{\gamma }\cdot a^{2}}{\eta_{s}}$$

where $Re_{P}$ is the Reynolds number (-) and $\rho_{s}$ is the density of the suspending medium (kg/m^3^).

It is shown that the apparent viscosity increases linearly with the increase in the shear strain rate in the case that inertia dominates viscous force, so the shear stress scales with the shear rate to a power of 2. Therefore, if this theory is applicable to highly flowable mortar and concrete, the quadratic term of shear rate in the modified Bingham model can be interpreted as an expression for particle momentum transfer.

#### 2.1.3. A More Reasonable Theory of Shear Thickening of Highly Flowable Mortar

A large number of studies [23,24,25] have shown that the thickness of the lubrication layer is about 2~5 mm in the pumping process. The particle radius of sand in the lubrication layer can be assumed to be 1 mm. The fluidity of the paste in the lubrication layer is very good. Assume that the density of the paste in the lubrication layer is 2000 kg/m^3^ and its viscosity is 0.01 Pa·s, which is ten times the viscosity of water. Even if the shear rate is as low as 5 s^−1^, the Reynolds number (*Re_P_*), calculated according to Equation (2), showed that the effect of particle inertia could not be ignored. Moreover, the shear rate during pumping is several orders of magnitude higher than 5 s^−1^.

However, study [26] showed that the quadratic relationship between shear stress and shear rate is not obtained by experiments even at very high Reynolds number for particle suspensions. In fact, when the rheological test results are treated with power functions, the maximum value of the index in the relationship between shear stress and shear rate never exceeds 1.58 [26]. This means that even at a high Reynolds number, the viscous force still has a significant influence on the rheological behavior of the material. Such experimental results obviously cannot be explained by grain inertia theory.

The quadratic relationship between shear stress and shear strain rate is also shown in practical cases, which mostly occur when solid particles are suspended in gas-phase fluid, which is obviously different from the rheological properties of highly flowable mortar and concrete [27]. If the modified Bingham model (Equation (3)) is used to represent the performance of the lubrication layer in the actual pumping process, and the rheological parameters measured by the rheometer at a low shear strain rate are used to evaluate the rheological behavior of the lubrication layer at a high shear strain rate during the pumping process, a high evaluation deviation is bound to occur. The evaluation deviation refers to the deviation between the fitting results according to the rheological experiment and the rheological behavior of concrete in the actual pumping engineering. The higher the shear strain rate, the greater the deviation of the evaluation. Therefore, the modified Bingham model, which contains the quadratic term of shear strain rate, is not suitable for the characterization of the rheological behavior of highly flowable mortar and concrete.

(3) $$\begin{array}{cc} \tau =\tau_{0}+\mu \cdot \dot{\gamma }+c\cdot {\dot{\gamma }}^{2} & \left(\tau \geq \tau_{0}\right)\\ \dot{\gamma }=0 & \left(0\leq \tau <\tau_{0}\right) \end{array}$$

where *τ* is yield stress (Pa); *μ* is coefficient of a first-order term in the shear strain rate (Pa·s); and *c* is the coefficient of a second-order term in the shear strain rate (Pa·s^2^).

Feys’ research [14] showed that cluster formation theory is more suitable to explain the shear thickening behavior of cementitious materials. The exponential form between shear stress and shear strain rate is more suitable to describe the shear thickening phenomenon of cementitious materials than the quadratic form. From a mathematical point of view, the quadratic term in the modified Bingham model is nothing more than a term in the Taylor expansion of the Herschel–Bulkley model (Equation (4)). At present, no research results have shown that the exponent of the Herschel–Bulkley model based on fresh cementitious materials can reach or exceed 2.

(4) $$\begin{array}{cc} \tau =\tau_{0}+K{\dot{\gamma }}^{n} & \left(\tau \geq \tau_{0}\right)\\ \dot{\gamma }=0 & \left(0\leq \tau <\tau_{0}\right) \end{array}$$

where *K* is the consistency coefficient (Pa·s*^n^*) and *n* is the consistency exponent (-).

Undeniably, there are still some problems in the exponential rheological model such as the dimension and the physical meaning of parameters. For example, although the dimensional problem of the non-Newtonian fluid model has not been effectively solved, it does not prevent the widespread application of this rheological model. Although these problems need to be further studied, the rheological model in an exponential form is more suitable for the characterization of the shear thickening properties of highly flowable mortar and concrete than the rheological model in a quadratic form according to the existing theories.

### 2.2. Couette Inverse Problem

A rheometer can be used to measure the rotational speed and torque of the fluid under constant rotational speed. There are many calculation methods to convert the rotational speed and torque into the rheological parameters of the fluid. However, it is the most reasonable method to use the solution of the Couette inverse problem to calculate the rheological parameters for fluids containing large particles such as highly flowable mortar and concrete [28,29]. For example, under the action of low shear stress, the rheological properties of fresh cementitious materials can often be characterized by the Bingham model. With the help of the Reiner–Riwlin equation (as shown in Equation (5), namely the solution of the Couette inverse problem based on the Bingham model) and the linear fitting method, two rheological parameters, yield stress and plastic viscosity, can be easily calculated [28].

(5) $$\Omega =\frac{1}{4\pi h\eta }\left(\frac{1}{R_{1}^{2}}-\frac{1}{R_{2}^{2}}\right)\cdot T-\frac{\tau_{0}}{\eta }\ln\frac{R_{2}}{R_{1}}$$

where Ω is rotational speed (rad/s); *T* is torque (N·m); *h* is effective height of the rotor (m); *η* is plastic viscosity (Pa·s); *τ*_0_ is yield stress (Pa); *R*_1_ is radius of rotor (m); and *R*_2_ is radius of outer cylinder (m).

At present, the Couette inverse problem based on the nonlinear rheological models has been well solved, which greatly facilitates the popularization and utilization of nonlinear rheological models. For example, the expression of the modified Bingham model is shown in Equation (3), and the solution of its Couette inverse problem is shown in Equation (6) [15]. The form of Equation (6) shows that it is difficult to calculate the rheological parameters of the modified Bingham model. More importantly, when fresh cementitious materials exhibit the behavior of shear thickening under high shear stress, the measured curves of rotational speed and torque are usually a very simple looking curve, which does not need to be represented by such a complex relation as Equation (6). This also indicates that the modified Bingham model is not suitable for the characterization of rheological properties of highly flowable mortar and concrete. In fact, up to now, no studies have shown that shear thickening behavior of fresh cementitious materials is characterized by the relation shown in Equation (6). In addition, there has never been a case in which Equation (6) has been used to calculate the rheological parameters of the modified Bingham model.

(6) $$\begin{array}{l} \Omega =\frac{\mu }{2c}\ln\frac{R_{1}}{R_{2}}+\frac{1}{2c}\cdot (\sqrt{\mu^{2}-4c\tau_{0}+4c\frac{T}{2\pi hR_{1}^{2}}}\\ -\sqrt{\mu^{2}-4c\tau_{0}+4c\frac{T}{2\pi hR_{2}^{2}}}\\ +\sqrt{4c\tau_{0}-\mu^{2}}\cdot \arctan\sqrt{\frac{\mu^{2}-4c\tau_{0}+4c\frac{T}{2\pi hR_{1}^{2}}}{4c\tau_{0}-\mu^{2}}}\\ -\sqrt{4c\tau_{0}-\mu^{2}}\cdot \arctan\sqrt{\frac{\mu^{2}-4c\tau_{0}+4c\frac{T}{2\pi hR_{2}^{2}}}{4c\tau_{0}-\mu^{2}}}) \end{array}$$

The expression of the Herschel–Bulkley model is shown in Equation (4), and the solution of its Couette inverse problem is shown in Equation (7) [15]. Equation (7) does not give the final expression of the solution of the Couette inverse problem. Reference [30] shows that Equation (7) has no solution in the real number range. Even if an approximate solution is given by numerical analysis, the calculation error caused by this method is difficult to characterize. For the actual pumping project, it is impossible for the construction workers to choose a rheological model that cannot accurately calculate the rheological parameters and is difficult to deal with the calculation error.

(7) $$\Omega =-\frac{n}{2}\left({\dot{\gamma }}_{2}-{\dot{\gamma }}_{1}\right)+\frac{n}{2}\int_{{\dot{\gamma }}_{1}}^{{\dot{\gamma }}_{2}}\frac{1}{1+\left(K/\tau_{0}\right){\dot{\gamma }}^{n}}d\dot{\gamma }$$

where ${\dot{\gamma }}_{1}$ is the shear strain rate on the fluid near the surface of the rotor (s^−1^) and ${\dot{\gamma }}_{2}$ is the shear strain rate on the fluid near the surface of the outer cylinder (s^−1^).

## 3. A Nonlinear Rheological Model for Highly Flowable Mortar

### 3.1. The New Rheological Model

A new rheological model, as shown in Equation (8), can be established:

(8) $$\begin{array}{cc} \sqrt[{n}]{\tau }-\sqrt[{n}]{\tau_{0}}=\kappa^{1/n}\dot{\gamma } & \left(\tau \geq \tau_{0}\right)\\ \dot{\gamma }=0 & \left(0\leq \tau <\tau_{0}\right) \end{array}$$

where *τ*_0_, *κ*, and *n* are three material parameters, *τ*_0_ being the yield stress (Pa), *κ* being the consistency coefficient (Pa·s*^n^*), and *n* being the consistency exponent (-). The value of *τ*_0_ is non-negative. The values of *κ* and *n* are always positive.

When the shear stress exceeds the yield stress, the differential viscosity *η_d_* of the fluid can be calculated by Equation (9) as follows:

(9) $$\eta_{d}=\frac{d\tau }{d\dot{\gamma }}=\frac{1}{d\dot{\gamma }/d\tau }=\frac{\kappa^{1/n}n}{\tau^{\frac{1}{n}-1}}$$

According to Equation (9), when the parameter *n* is higher than 1, the larger the value of *n*, the more obvious the shear thickening effect. When the parameter *n* is in the range from 0 to 1, the smaller the value of *n*, and the more obvious the shear thinning effect. When the value of *n* equals 1, this model becomes the Bingham model.

### 3.2. The Solution of the Couette Inverse Problem

According to Equation (9) in the literature [15], the following integral expression (Equation (10)) can be used to solve the Couette inverse problem based on the nonlinear rheological model when a coaxial cylinder rheometer is used.

(10) $$\Omega =-\frac{1}{2}\int_{\tau_{1}}^{\tau_{2}}\frac{1}{\tau }\cdot \dot{\gamma }d\tau$$

where *τ*_1_ is the shear stress on the fluid near the surface of the rotor (Pa) and *τ*_2_ is the shear stress on the fluid near the surface of the outer cylinder (Pa).

Replacing Equation (10) by means of Equation (8) when the shear stress is no less than the yield stress, the following relationship (Equation (11)) is gained.

(11) $$\begin{array}{l} \Omega =-\frac{1}{2}\int_{\tau_{1}}^{\tau_{2}}\frac{1}{\tau }\cdot \frac{1}{\kappa^{1/n}}\left(\tau^{1/n}-\tau_{0}^{1/n}\right)d\tau \\ =-\frac{n}{2\kappa^{1/n}}\left(\tau_{2}^{1/n}-\tau_{1}^{1/n}\right)+\frac{\tau_{0}^{1/n}}{2\kappa^{1/n}}\ln\frac{\tau_{2}}{\tau_{1}} \end{array}$$

The value of the shear stress can be calculated according to Equation (12) when a coaxial cylinder rheometer is used [1,6,12].

(12) $$\tau =\frac{T}{2\pi hr^{2}}$$

Therefore, the value of *τ*_1_ and *τ*_2_ can be calculated as follows Equations (13) and (14) [6].

(13) $$\tau_{1}=\frac{T}{2\pi hR_{1}^{2}}$$

(14) $$\tau_{2}=\frac{T}{2\pi hR_{2}^{2}}$$

Replacing Equation (11) by means of Equations (13) and (14), the following relationship Equation (15) can be gained.

(15) $$\Omega =\frac{n}{2\cdot {\left(2\pi h\kappa \right)}^{1/n}}\left(R_{1}^{-2/n}-R_{2}^{-2/n}\right)\cdot T^{1/n}-{\left(\frac{\tau_{0}}{\kappa }\right)}^{1/n}\ln\frac{R_{2}}{R_{1}}$$

Equation (15) is the solution of the Couette inverse problem based on the new rheological model Equation (8).

Equation (15) indicates that the solution of the Couette inverse problem of the new rheological model (Equation (8)) has a clear analytical solution and a simple form of analytical solution.

### 3.3. Comparison Among Other Rheological Models

Equation (8) maintains the form of an exponential model. In order to deepen the understanding of the new rheological model, a comparison of the relevant exponential rheological models was made.

The simplest nonlinear rheological model is the non-Newtonian fluid model. Its expressions are shown in Equations (16) or (17). The two expressions, which are different in form but essentially the same, are given here to facilitate the comparison of rheological models. The model can be used to characterize the shear thickening or shear thinning properties of fluids without yield stress. The solution of the Couette inverse problem is shown in Equation (18).

(16) $$\tau =\eta {\dot{\gamma }}^{n}$$

(17) $$\sqrt[{n}]{\tau }=\eta^{1/n}\dot{\gamma }$$

where *η* is the viscosity (Pa·s).

(18) $$\Omega =\frac{n}{2\cdot {\left(2\pi h\eta \right)}^{1/n}}\left(R_{1}^{-2/n}-R_{2}^{-2/n}\right)\cdot T^{1/n}$$

When the shear stress exceeds the critical shear stress, highly flowable mortar will show the performance of shear thickening. However, highly flowable mortar has yield stress, so it is necessary to introduce a yield stress on the basis of the non-Newtonian fluid model. The Herschel–Bulkley model (Equation (4)) introduces a yield stress based on Equation (16), while the new rheological model established in this paper introduces a yield stress based on Equation (17). The two approaches may not seem very different, but the Couette inverse problem based on the Herschel–Bulkley model has no solution in the real number range. It has no influence on the theoretical research, but causes great difficulties for the calculation of rheological parameters in practical engineering.

In the solution of the Couette inverse problem of the new rheological model (Equation (15)), the rotational speed can be obviously divided into two terms. One related to the torque corresponds exactly to the solution of the Couette inverse problem based on the non-Newtonian fluid model and another that is independent of the torque corresponds exactly to the contribution of the yield stress to the rotational speed. This method of establishing a rheological model only changes the way of introducing a yield stress. This small change not only maintains the advantage of the exponential rheological model in characterizing shear thickening or shear thinning properties, but also benefits the calculation of rheological parameters.

## 4. Materials and Experimental Methods

### 4.1. Raw Materials

**Figure 2:**
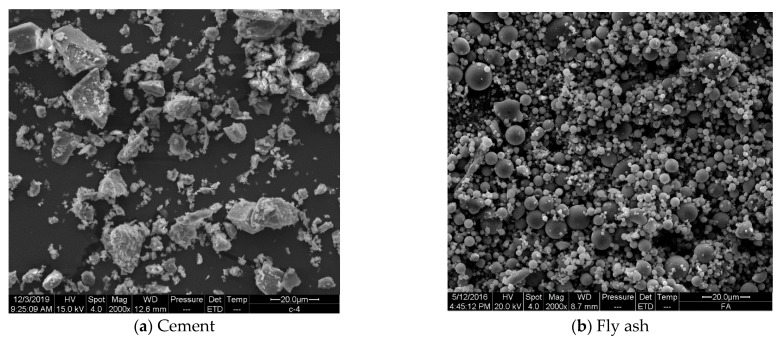

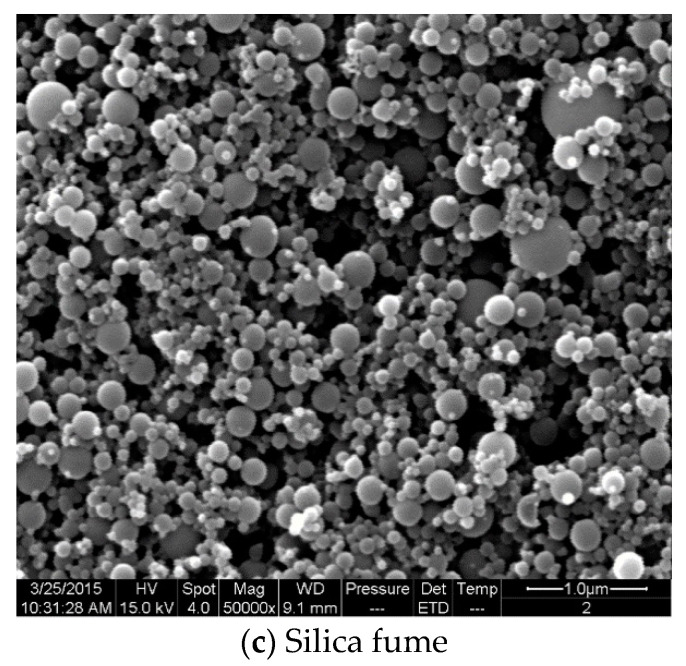
Particle morphology of the three cementitious materials. (**a**) Particle morphology of Cement; (**b**) Particle morphology of FA; (**c**) Particle morphology of SF.

PI 42.5 cement (Cement), Class II fly ash (FA), and silica fume (SF) were utilized. The specific surface area of cement and silica fume was 355 m^2^/kg (Blaine) and 23.37 m^2^/g (BET), respectively. The 45 μm square hole screen residual of fly ash was 31.7%. The chemical composition of the three cementitious materials is shown in Table 1. The microscopic morphology of the three cementitious materials is shown in Figure 2. ISO standard sand was used as the fine aggregate to ensure the stability of rheological test results. A polycarboxylic superplasticizer (SP) with 58.70% solid content was utilized.

### 4.2. Mix Proportion

The majority of studies [23,24,25] showed that the composition and performance of the lubrication layer formed during the pumping process are very similar to that of the component mortar of pumped concrete. Table 2 shows the mix proportions of the mortars. The control mortar, M0, refers to the mix proportion of component mortar of concrete with a strength grade of C50. Its water:binder ratio (W/B) was 0.25, the sand:binder ratio (SBR) was 1.37, the volume fraction of fly ash and silica fume in the cementitious material were 42.51%, 7.18%, respectively, and the dosage of SP was 2.70%. The mix proportions of testing mortars were determined by changing the sand:binder ratio, the content of fly ash and silica fume in the cementitious material, and the dosage of SP according to the single factor control method. The fluidity of M0 measured by the table jumping test was 284 mm.

### 4.3. Mortar Preparation

In order to minimize the effect of mixing process on the rheological properties of mortar and ensure a fully homogeneous mix, the mixing procedure was strictly controlled before the rheological experiment as follows.

Pour the weighed cement, FA and SF into the mixing bucket and mix them slowly with a hand-held mixer for 1 min. After mixing water and SP evenly, add it into the cementitious material and mix it slowly for 0.5 min. After adding the fine aggregate, mix it slowly for 0.5 min, then stir it quickly for 6 min, then begin the rheological test.

### 4.4. Rheometer and Protocol

A coaxial cylinder rheometer was utilized. The diameter of the outer cylinder and inner cylinder were 0.164 m and 0.128 m, respectively. The effective height of the inner cylinder was 0.128 m. During the rheological experiment, the inner cylinder was stationary and the outer cylinder rotated. There was a suitable number of ribs on the wall of the outer cylinder and inner cylinder, which ensured that there was no slippage between the mortar and the surface of the rheometer cylinder during the rheological experiment.

**Figure 3:**
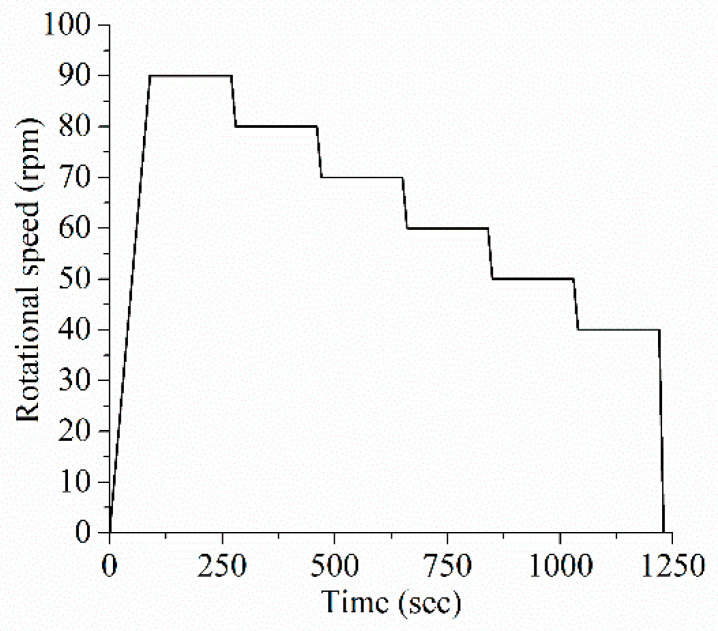
Rheological protocol.

The rheological protocol adopted in this study is shown in Figure 3.

## 5. Results and Discussion

### 5.1. Relationship between Rotational Speed and Torque

**Figure 4:**
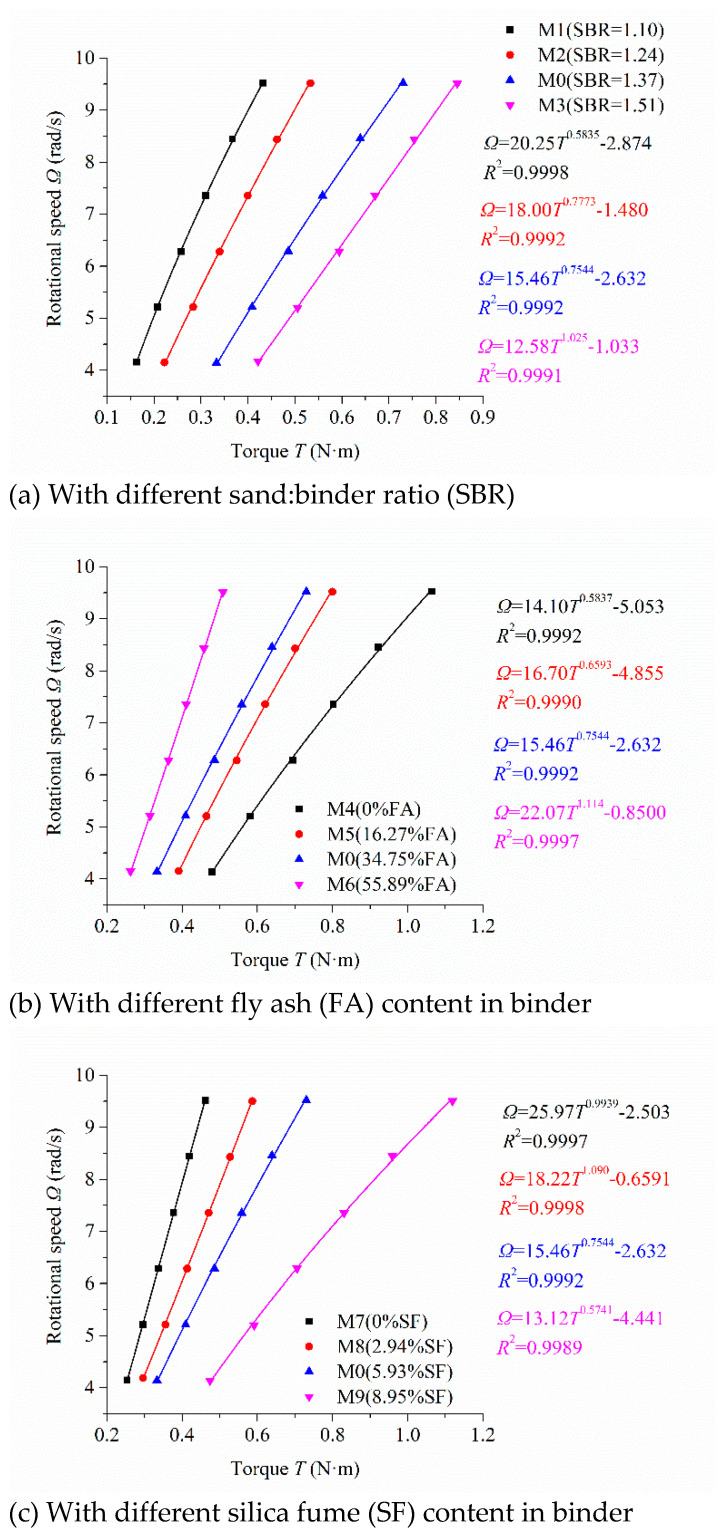

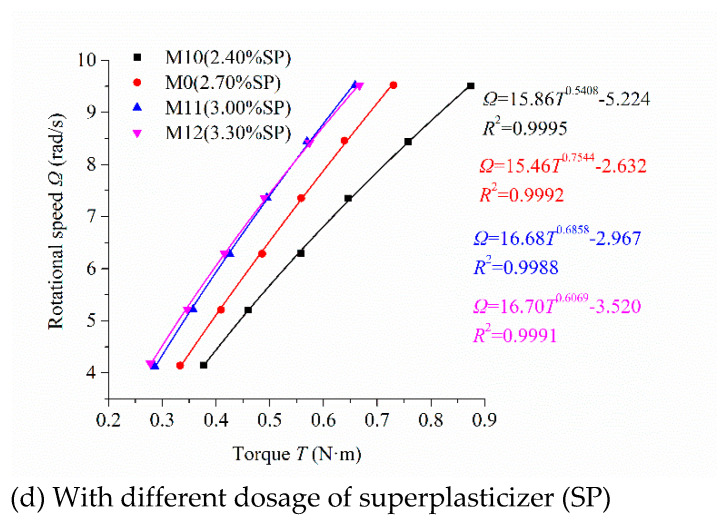
Relationship between the rotational speed and torque of mortars fitted by the new rheological model.

According to the rheological protocol described in Figure 3, the scatters of rotational speed Ω and torque *T* of mortar are obtained under the steadily flowing state [31] (Figure 4). The analytical formula (Equation (15)) was used to conduct nonlinear fitting and iterative calculation for the scatters in Figure 4. The fitting results are also shown in Figure 4. The correlation coefficient *R*^2^ was very close to 1 for all the fitting results.

Figure 4a describes the relationship between the rotational speed and torque of the mortars with different sand:binder ratios. The torque increased with the increase in the sand:binder ratio under the same rotational speed. Figure 4b reveals the influence of fly ash content on the relationship between the rotational speed and torque of mortars. The torque decreased with the increase in fly ash content under the same rotational speed. Figure 4c shows the influence of silica fume content on the relationship between the rotational speed and torque of mortars. The torque increased continuously with the increase in silica fume content under the same rotational speed. Figure 4d displays the relationship between the rotational speed and torque of mortars under different SP dosage. The torque decreased continuously with the increase in the dosage of SP at the same rotational speed. In Figure 4d, the scatters and the fitting curves of M11 and M12 almost coincided because of the saturating dosage of SP. These results have also been investigated by other researchers [5,32].

### 5.2. Determination of Three Parameters in the New Rheological Model

In order to further study and analyze the rheological behavior of mortars, three rheological parameters of the newly established nonlinear rheological model need to be calculated. According to the fitting relation between the rotational speed and torque of mortars in Figure 4 and Equation (15), three rheological parameters were calculated by solving nonlinear equations. Table 3 shows the calculated results.

Lines 2 through 5 in Table 3 show the variation rules of yield stress *τ*_0_, consistency coefficient *κ*, and consistency exponent *n* of mortars with different sand:binder ratios. With the increase in the sand:binder ratio, the percentage of sand increased, the yield stress *τ*_0_ and the consistency coefficient *κ* of mortars increased, and the consistency exponent *n* of mortars decreased. The consistency exponent *n* of mortars M0, M1, and M2, whose sand:binder ratio was less than 1.4, was significantly higher than 1. These mortars showed obvious shear thickening. The consistency exponent *n* of mortar M3, whose sand:binder ratio was higher than 1.4, was approximately equal to 1. It did not show an obvious shear thickening phenomenon. Mortars showing the performance of shear thickening were mainly due to the paste containing Brownian particles (i.e., cementitious materials). These particles form clusters in the paste [14,33,34,35,36] to increase its consistency. Sand itself has no performance of shear thickening. Therefore, the content of paste decreases and its viscosity increases with the increase in the sand:binder ratio and results in the performance of shear thickening of mortar being less obvious.

Lines 6 through 9 in Table 3 show the influence of fly ash content in binder on the yield stress *τ*_0_, consistency coefficient *κ*, and consistency exponent *n* of mortars. With the increase of fly ash content in the binder, the yield stress *τ*_0_ of mortar decreased continuously, the consistency coefficient *κ* increased continuously, and the consistency exponent *n* decreased continuously. Fly ash is composed mainly of spherical glassy particles (Figure 2b) and forms less clusters than cement, which are composed mainly of multangular particles (Figure 2a) [14,36,37]. The consistency exponent *n* of mortars M4, M5, and M0, whose volume fractions of FA were less than 50%, was higher than 1, indicating their shear thickening. The more volume fraction of FA in the binder, the less obvious the shear thickening of the mortar. Even mortar M6, whose volume fraction of FA was higher than 50%, illustrated slight shear thinning when its consistency exponent *n* decreased to less than 1.

Lines 10 through 13 in Table 3 reveal the influence of silica fume content in binder on yield stress *τ*_0_, consistency coefficient *κ*, and consistency exponent *n* of mortar. With the increase in the silica fume content, the yield stress *τ*_0_ and consistency exponent *n* of mortar initially decreased and subsequently increased, and the consistency coefficient *κ* of the mortar initially increased and subsequently decreased. The consistency exponent *n* of mortar M7 was approximately equal to 1, indicating that mortar M7 did not show obvious shear thickening.

Spherical silica fume particles have a much smaller particle size than cement and fly ash particles (Figure 2). A suitable volume of silica fume fills in the space among the cement and fly ash particles to increase the density of the mixture and enlarge the particle size distribution. This can improve the lubricating effect and decrease the inner friction force in the mixture. Therefore, the consistency exponent *n* of mortar M8 reached a much lower value, less than 1, to illustrate aa shear thinning phenomenon. The high volume of silica fume increased the surface area of the mixture to enlarge the water demand of the mixture. The consistency of mortar increased at the same water:binder ratio and its deformation resistance also increased. Therefore, the greater the silica fume content in cementitious materials, the more Brownian particles in the same volume, which will increase the degree of shear thickening. The consistency exponent *n* of mortar M0 and M9 increased gradually with the volume fractions of SF in binder to show an obvious shear thickening.

Lines 14 through 17 in Table 3 display the effect of the dosage of SP on the yield stress *τ*_0_, consistency coefficient *κ*, and consistency exponent *n* of mortar. With the increase in the dosage of SP, the yield stress *τ*_0_ of mortar continuously decreased, the consistency coefficient *κ* initially increased and subsequently decreased, and the consistency exponent *n* initially decreased and subsequently increased. The consistency exponent *n* of mortars M10, M11, M0, and M12 was much higher than 1, manifesting their obvious shear thickening. The consistency exponent *n* of mortar M10 was the largest among the four mortars, indicating that its shear thickening degree was the largest.

### 5.3. Relationship between Shear Stress and Shear Strain Rate

Although the consistency exponent *n* in the new rheological model can reflect the shear thickening performance of mortar, it is only judged according to a parameter in the new rheological model. Whether the new rheological model can correctly represent the performance of the shear thickening of mortar needs to be verified by unanimously approved indicators. The relationship between shear stress and shear strain rate can directly reflect the performance of shear thickening. According to Table 3 and Equation (8), the relationship between shear stress and shear strain rate were calculated and shown in Table 4.

**Figure 5:**
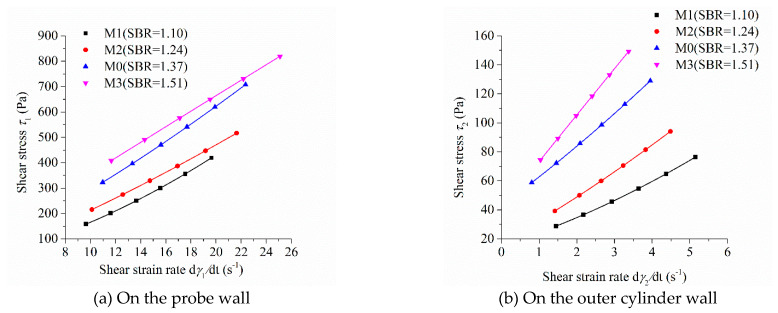
Relationship between shear stress and shear strain rate of mortars with different sand-binder ratios.

**Figure 6:**
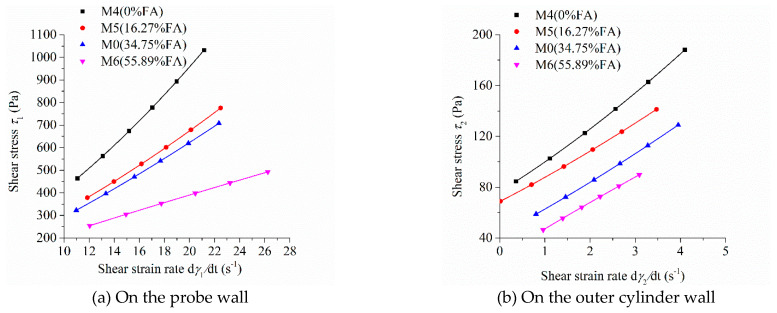
Relationship between shear stress and shear strain rate of mortars with different fly ash (FA) contents.

**Figure 7:**
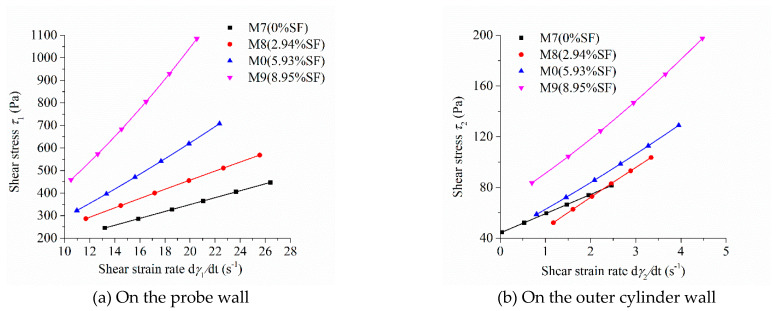
Relationship between shear stress and shear strain rate of mortars with different silica fume (SF) contents.

**Figure 8:**
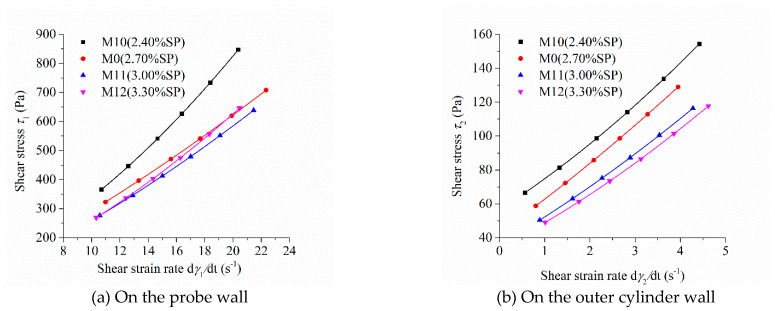
Relationship between the shear stress and shear strain rate of mortars with different dosages of superplasticizer (SP).

As the shear stress and shear strain rate at different positions in the measured cylinder are different during the rheological test, the image of “shear stress-shear rate” should indicate the location of the measured point in the rheometer. Figure 5, Figure 6, Figure 7 and Figure 8 show the relationship between shear stress and shear strain rate on the probe wall and the outer cylinder wall, respectively. According to Equation (12), when the torque is unchanged, the shear stress on the probe wall is the largest, while the shear stress on the outer cylinder wall is the smallest. Therefore, Figure 5a, Figure 6a, Figure 7a and Figure 8a show the relationship between shear stress and shear strain rate in the case of high shear stress. Figure 5b, Figure 6b, Figure 7b and Figure 8b show the relationship between shear stress and shear strain rate in the case of low shear stress. It shows from Figure 5b, Figure 6b, Figure 7b and Figure 8b and Table 4 that even the lowest shear stress was higher than the yield stress of the mortars. This indicates that the measured data during the rheological experiment were not affected by the plug flow.

Most of the mortars showed the performance of shear thickening, which can be determined from the bending direction of the curve (Figure 5, Figure 6, Figure 7 and Figure 8), aside from the power index of shear stress (Table 4).

Figure 5 shows the relationship between shear stress and shear strain rate of mortars at different sand:binder ratios. The shear stress of mortar at the same shear strain rate increased continuously with the increase in the sand:binder ratio, which was consistent with the changing tendency of the rotational speed of mortar at the same torque in Figure 4a. The upwardly bending degree of the curve of “shear stress-shear strain rate” of M1 was the largest among the mortars M0 to M3. Therefore, the shear thickening degree of mortar M1 was the largest, which was consistent with the largest consistency exponent *n* of mortar M1 in lines 2 through 5 in Table 3. The shear stress–shear strain rate curve of mortar M3 was approximately a straight line. This means that M3 had little shear thickening performance, which is consistent with its consistency exponent *n*, which was close to 1.

Figure 6 exhibits the influence of fly ash content on the relationship between shear stress and shear strain rate of mortars. The shear stress of mortar at the same shear strain rate and the upwardly bending degree of the curve of “shear stress-shear strain rate” of mortars decreased continuously with the increase in fly ash content, which is consistent with the changing tendency of the rotational speed of mortar at the same torque in Figure 4b. The upwardly bending degree of the curve of “shear stress-shear strain rate” of M4 was the largest among the mortars M0, M4, M5, and M6. Therefore, the shear thickening degree of mortar M4 was the largest, which is consistent with the result that the consistency exponent *n* of mortar M4 was the largest in lines 6 through 9 in Table 3. The shear stress–shear strain rate curve of mortar M6 was a downwardly bending line. This means that M6 had a little shear thinning performance, which is consistent with the consistency exponent *n* of M6 that was less than 1.

Figure 7 reveals the influence of silica fume content on the relationship between shear stress and shear strain rate. The shear stress at the same shear strain rate increased continuously with the increase in silica fume content, which was consistent with the changing tendency of the rotational speed of the mortar at the same torque in Figure 4c. The shear stress–shear strain rate curve of mortar M7 and M8 were approximately straight lines. This means that M7 and M8 had little shear thickening or shear thinning performance, which is consistent with the consistency exponent *n* of M7 and M8 that was close to 1. The upwardly bending degree of the curve of “shear stress-shear strain rate” of M9 was the largest among the M0, M7, M8, and M9 mortars. This means that the shear thickening degree of mortar M9 was the largest, being consistent with its largest consistency exponent *n* in lines 10 through 13 in Table 3.

Figure 8 displays the relationship between the shear stress and shear strain rate of mortar with different dosages of SP. The shear stress under the same shear strain rate decreased continuously with the increase in SP content, which is consistent with the changing tendency of the rotational speed of mortar at the same torque in Figure 4d. The upwardly bending degree of the “shear stress–shear strain rate” curve of the four mortars was obvious. They showed obvious shear thickening performance, which is consistent with their consistency exponent *n*, which was much greater than 1 in Table 3. The bending degree of the curve of the “shear stress–shear strain rate” of M10 was the largest among the mortars M0, M10, M11, and M12. This means that the shear thickening degree of mortar M10 was the largest, being consistent with its largest consistency exponent *n* in lines 14 through 17 in Table 3.

### 5.4. Relationship between Differential Viscosity and Shear Stress of Mortars

**Figure 9:**
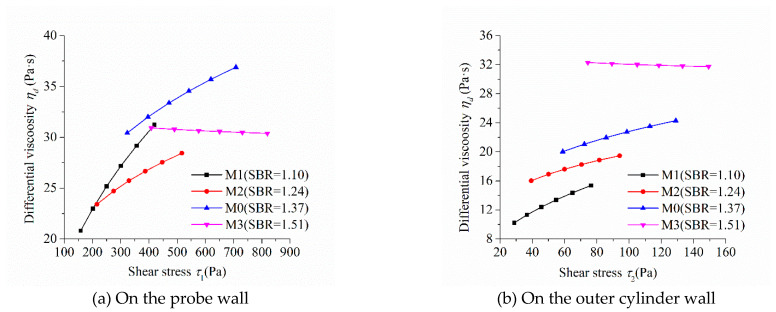
Relationship between thee differential viscosity and shear stress of mortars with different sand:binder ratios.

Differential viscosity can intuitively characterize the performance of the shear thickening of mortar. Figure 9 shows the relationship between the differential viscosity and the shear stress of mortars with different sand:binder ratios. The differential viscosity of mortars M1, M2, and M0 obviously increased with the increase in shear stress and M1 had the highest growth rate. This means that these mortars had an obvious performance of shear thickening and M1 was the most significant. The differential viscosity of M3 changed little with the increase in shear stress and means that M3 showed little shear thickening performance, which is consistent with the previously discussed result.

**Figure 10:**
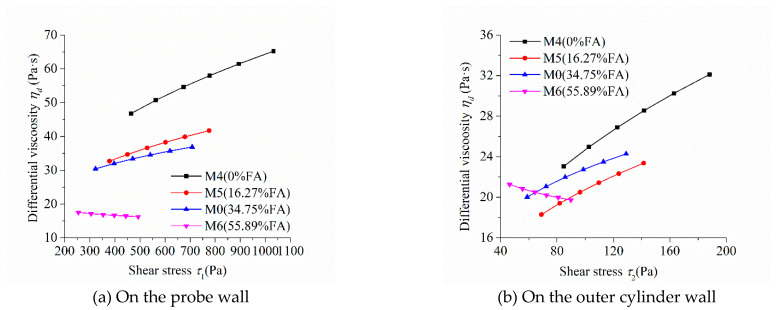
Relationship between the differential viscosity and shear stress of mortars with different FA contents.

Figure 10 shows the influence of fly ash content on the relationship between the differential viscosity and the shear stress of mortars. The differential viscosity of M4, M5, and M0 increased continuously with the increase in shear stress, while that of M6 decreased slightly. This indicates that mortars M4, M5, and M0 showed a performance of shear thickening, while M6 showed a performance of shear thinning, which was consistent with the previously discussed results. The differential viscosity of M4 containing no fly ash in its binder had the highest growth rate with the increase in shear stress, indicating that the shear thickening performance of M4 was the most significant. Therefore, fly ash in the binder can reduce the shear thickening performance of cementitious materials and enhance their flow characteristics.

**Figure 11:**
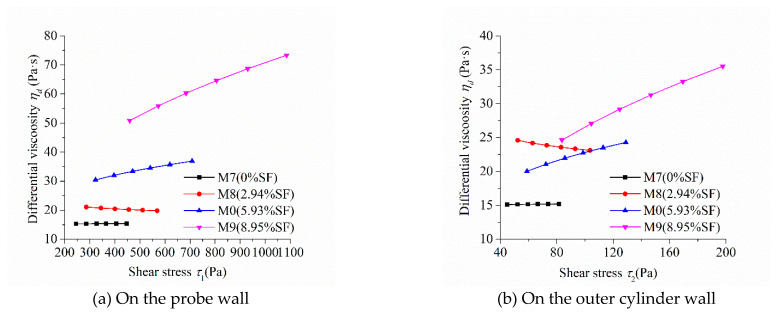
Relationship between the differential viscosity and shear stress of mortars with different SF contents.

Figure 11 exhibits the influence of silica fume content on the relationship between the differential viscosity and shear stress of mortars. The differential viscosity of M0 and M9 containing a high volume of silica fume increased continuously with the increase in shear stress, while that of M7 and M8 containing none or a small volume of silica fume remained unchanged or decreased slightly. It suggests that M0 and M9 showed an obvious shear thickening performance, while M7 and M8 showed no shear thickening or slight shear thinning, which is consistent with the previously discussed results. The differential viscosity of M9 containing the highest volume of silica fume had the highest growth rate and M8 containing a suitable volume of silica fume showed a minus growth rate with the increase in shear stress. This means that the shear thickening performance of M9 was the most significant and M8 had a shear shinning performance. Therefore, silica fume can reduce or increase the shear thickening performance of cementitious materials and enhance or deteriorate their flow characteristics based on its dosage.

**Figure 12:**
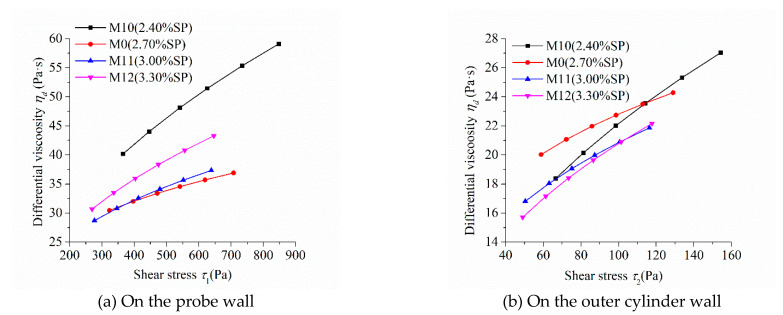
Relationship between the differential viscosity and shear stress of mortars with different dosages of SP.

Figure 12 reveals the relationship between the differential viscosity of mortar with different dosages of SP and shear stress. The differential viscosity of mortars M10, M0, M11, and M12 increased continuously with the increase in shear stress, indicating that these mortars showed an obvious performance of shear thickening. This was consistent with the previously discussed result. The differential viscosity of mortar M10, whose SP dosage was the lowest, had the highest growth rate with the increase in shear stress, indicating that the shear thickening performance of M10 was the most significant among mortars M10, M0, M11, and M12. The growth rate of differential viscosity of mortar M12, whose SP dosage was the highest, was higher than that of M0 and M11, whose SP dosage was less than M12, indicating that the shear thickening performance of M12 was more obvious than M0 and M11. The addition of SP can improve the fluidity of cementitious materials. As a result, their shear thickening performance reduces. However, an excessive dosage of SP may result in heterogeneity of mixture to enhance the shear thickening performance.

## 6. Conclusions

A new exponential rheological model comprising of three parameters, yield stress, consistency coefficient, and consistency exponent, was established. The analytical solution of its Couette inverse problem can be easily obtained to benefit the calculation of rheological parameters. The rheological parameters of this model can quantitatively characterize the shear thickening behavior of highly flowable cementitious materials.

The calculating results of the rheological parameters of the newly established model for the mortars with different compositions showed that the consistency exponent of mortar decreased with the increase of its sand:binder ratio or the dosage of fly ash in the binder. This indicates that the shear thickening degree of the mortar decreases. The consistency exponent of mortar initially decreased and subsequently increased with the increase in silica fume content or the dosage of superplasticizer. This illustrates that the degree of shear thickening of mortar initially decreases and subsequently increases. These varying patterns were confirmed by the rheological experiment of mortars.

## Figures and Tables

**Table 1:** Chemical compositions of three cementitious materials analyzed by x-ray fluorescence (XRF)/%.

Composition	Cement	FA	SF
SiO_2_	15.73	46.67	96.36
Al_2_O_3_	3.31	34.66	0.33
Fe_2_O_3_	4.14	6.13	0.05
CaO	70.36	6.28	0.58
MgO	1.38	0.81	0.34
SO_3_	3.47	0.80	1.05
Na_2_O_eq_	0.55	1.19	0.86
Total	98.94	96.54	99.57

Note: Na_2_O_eq_ includes Na_2_O and 0.658K_2_O.

**Table 2:** The mix proportions of the mortars.

Sample	Composition of Binders/%			SBR	W/B	SP/%
	Cement	FA	SF			
M0 (Control group)	59.32	34.75	5.93	1.37	0.25	2.70
M1 (SBR = 1.10)	59.32	34.75	5.93	1.10	0.25	2.70
M2 (SBR = 1.24)	59.32	34.75	5.93	1.24	0.25	2.70
M3 (SBR = 1.51)	59.32	34.75	5.93	1.51	0.25	2.70
M4 (0% FA)	90.91	0.00	9.09	1.37	0.25	2.70
M5 (16.27% FA)	76.12	16.27	7.61	1.37	0.25	2.70
M6 (55.89% FA)	40.10	55.89	4.01	1.37	0.25	2.70
M7 (0% SF)	63.10	36.90	0.00	1.37	0.25	2.70
M8 (2.94% SF)	61.24	35.81	2.94	1.37	0.25	2.70
M9 (8.95% SF)	57.45	33.60	8.95	1.37	0.25	2.70
M10 (2.40% SP)	59.32	34.75	5.93	1.37	0.25	2.40
M11 (3.00% SP)	59.32	34.75	5.93	1.37	0.25	3.00
M12 (3.30% SP)	59.32	34.75	5.93	1.37	0.25	3.30

Note: SBR is the sand:binder ratio, W/B is the water:binder ratio, and SP is the proportion of polycarboxylic superplasticizer to the total amount of cementitious materials.

**Table 3:** Three parameters of mortars with different mix proportions.

Row Number	Sample	Yield Stress *τ*_0_ (Pa)	Consistency Coefficient *κ* (Pa·s^n^)	Consistency Exponent *n* (-)
2	M1 (SBR = 1.10)	15.63	1.94	1.71
3	M2 (SBR = 1.24)	18.25	8.96	1.29
4	M0 (SBR = 1.37)	43.32	9.71	1.33
5	M3 (SBR = 1.51)	40.81	33.79	0.98
6	M4 (0% FA)	76.54	3.62	1.71
7	M5 (16.27% FA)	68.72	4.90	1.52
8	M0 (34.75% FA)	43.32	9.71	1.33
9	M6 (55.89% FA)	25.34	25.38	0.90
10	M7 (0% SF)	44.22	14.94	1.01
11	M8 (2.94% SF)	22.41	28.34	0.92
12	M0 (5.93% SF)	43.32	9.71	1.33
13	M9 (8.95% SF)	67.19	3.78	1.74
14	M10 (2.40% SP)	56.63	1.98	1.85
15	M0 (2.70% SP)	43.32	9.71	1.33
16	M11 (3.00% SP)	36.20	5.86	1.46
17	M12 (3.30% SP)	34.22	3.30	1.65

**Table 4:** Rheological expression of shear stress and shear strain rate.

Row Number	Sample	Rheological Expression
2	M1 (SBR = 1.10)	$\{\begin{array}{cc} \tau^{0.5835}-15.63^{0.5835}=1.473\dot{\gamma } & \left(\tau \geq 15.63\mathrm{Pa}\right)\\ \dot{\gamma }=0 & \left(0\leq \tau <15.63\mathrm{Pa}\right) \end{array}$
3	M2 (SBR = 1.24)	$\{\begin{array}{cc} \tau^{0.7773}-18.25^{0.7773}=5.500\dot{\gamma } & \left(\tau \geq 18.25\mathrm{Pa}\right)\\ \dot{\gamma }=0 & \left(0\leq \tau <18.25\mathrm{Pa}\right) \end{array}$
4	M0 (SBR = 1.37)	$\{\begin{array}{cc} \tau^{0.7544}-43.32^{0.7544}=5.554\dot{\gamma } & \left(\tau \geq 43.32\mathrm{Pa}\right)\\ \dot{\gamma }=0 & \left(0\leq \tau <43.32\mathrm{Pa}\right) \end{array}$
5	M3 (SBR = 1.51)	$\{\begin{array}{cc} \tau^{1.026}-40.81^{1.026}=36.96\dot{\gamma } & \left(\tau \geq 40.81\mathrm{Pa}\right)\\ \dot{\gamma }=0 & \left(0\leq \tau <40.81\mathrm{Pa}\right) \end{array}$
6	M4 (0% FA)	$\{\begin{array}{cc} \tau^{0.5837}-76.54^{0.5837}=2.119\dot{\gamma } & \left(\tau \geq 76.54\mathrm{Pa}\right)\\ \dot{\gamma }=0 & \left(0\leq \tau <76.54\mathrm{Pa}\right) \end{array}$
7	M5 (16.27% FA)	$\{\begin{array}{cc} \tau^{0.6593}-68.72^{0.6593}=2.852\dot{\gamma } & \left(\tau \geq 68.72\mathrm{Pa}\right)\\ \dot{\gamma }=0 & \left(0\leq \tau <68.72\mathrm{Pa}\right) \end{array}$
8	M0 (34.75% FA)	$\{\begin{array}{cc} \tau^{0.7544}-43.32^{0.7544}=5.554\dot{\gamma } & \left(\tau \geq 43.32\mathrm{Pa}\right)\\ \dot{\gamma }=0 & \left(0\leq \tau <43.32\mathrm{Pa}\right) \end{array}$
9	M6 (55.89% FA)	$\{\begin{array}{cc} \tau^{1.114}-25.34^{1.114}=36.68\dot{\gamma } & \left(\tau \geq 25.34\mathrm{Pa}\right)\\ \dot{\gamma }=0 & \left(0\leq \tau <25.34\mathrm{Pa}\right) \end{array}$
10	M7 (0% SF)	$\{\begin{array}{cc} \tau^{0.9938}-44.32^{0.9938}=14.69\dot{\gamma } & \left(\tau \geq 44.22\mathrm{Pa}\right)\\ \dot{\gamma }=0 & \left(0\leq \tau <44.22\mathrm{Pa}\right) \end{array}$
11	M8 (2.94% SF)	$\{\begin{array}{cc} \tau^{1.090}-22.41^{1.090}=38.35\dot{\gamma } & \left(\tau \geq 22.41\mathrm{Pa}\right)\\ \dot{\gamma }=0 & \left(0\leq \tau <22.41\mathrm{Pa}\right) \end{array}$
12	M0 (5.93% SF)	$\{\begin{array}{cc} \tau^{0.7544}-43.32^{0.7544}=5.554\dot{\gamma } & \left(\tau \geq 43.32\mathrm{Pa}\right)\\ \dot{\gamma }=0 & \left(0\leq \tau <43.32\mathrm{Pa}\right) \end{array}$
13	M9 (8.95% SF)	$\{\begin{array}{cc} \tau^{0.5741}-67.19^{0.5741}=3.312\dot{\gamma } & \left(\tau \geq 67.19\mathrm{Pa}\right)\\ \dot{\gamma }=0 & \left(0\leq \tau <67.19\mathrm{Pa}\right) \end{array}$
14	M10 (2.40% SP)	$\{\begin{array}{cc} \tau^{0.5408}-56.63^{0.5408}=1.446\dot{\gamma } & \left(\tau \geq 56.63\mathrm{Pa}\right)\\ \dot{\gamma }=0 & \left(0\leq \tau <56.63\mathrm{Pa}\right) \end{array}$
15	M0 (2.70% SP)	$\{\begin{array}{cc} \tau^{0.7544}-43.32^{0.7544}=5.554\dot{\gamma } & \left(\tau \geq 43.32\mathrm{Pa}\right)\\ \dot{\gamma }=0 & \left(0\leq \tau <43.32\mathrm{Pa}\right) \end{array}$
16	M11 (3.00% SP)	$\{\begin{array}{cc} \tau^{0.6858}-36.20^{0.6858}=3.363\dot{\gamma } & \left(\tau \geq 36.20\mathrm{Pa}\right)\\ \dot{\gamma }=0 & \left(0\leq \tau <36.20\mathrm{Pa}\right) \end{array}$
17	M12 (3.30% SP)	$\{\begin{array}{cc} \tau^{0.6069}-34.22^{0.6069}=2.064\dot{\gamma } & \left(\tau \geq 34.22\mathrm{Pa}\right)\\ \dot{\gamma }=0 & \left(0\leq \tau <34.22\mathrm{Pa}\right) \end{array}$

## Data Availability

Data sharing not applicable.

## Sample Availability

Samples of compounds are not available from the authors.
